# Supplementary material for: A DNA barcode library for Culex mosquitoes (Diptera: Culicidae) of South America with the description of two cryptic species of subgenus Melanoconion
Source: PLoS One. 2025 Feb 21;20(2):e0310571. doi: 10.1371/journal.pone.0310571 (PMC11845035; doi:10.1371/journal.pone.0310571)
Supplement: S2 Table — Species are listed alphabetically by subgenus. (DOCX) [file pone.0310571.s002.docx]

**S1 Table List of the *Culex* species corresponding to the voucher specimens that were COI sequenced in this study.** Species are listed alphabetically by subgenus and the life stage is indicated for each taxa (M: male with dissected genitalia; F: female; L: larva).

| **Species** | **Voucher specimen** | **Life stage** |
| --- | --- | --- |
| **Subgenus *Aedinus*** | | |
| *Culex accelerans* Root, 1927 | MB20245, ST11346 | M |
| *Culex amazonensis* (Lutz, 1905) | MB20287, MB20292 | M |
| **Subgenus *Anoedioporpa*** | | |
| *Culex originator* Gordon & Evans, 1922 | MB10810, MB10811, ST10667, ST10945, ST11150 | M/L |
| **Subgenus *Carrollia*** | | |
| *Culex antunesi* Lane & Whitman, 1943 | ST10855, ST10868, ST10869 | M/F/L |
| *Culex bonnei* Dyar, 1921 | ST11427, ST11428 | L |
| *Culex secundus* Bonne-Wepster & Bonne, 1920 | MB10840, ST10257, ST10258 | M/F/L |
| *Culex urichii* (Coquillett, 1906) | MB10038, MB10039, ST10175, ST10188, ST10194 | M/F/L |
| **Subgenus *Culex*** | | |
| *Culex bonneae* Dyar & Knab, 1919 | ST11379, ST11380, ST11392, ST11393 | M/L |
| *Culex brevispinosus* Bonne-Wepster & Bonne, 1920 | ST11383, ST11384 | M |
| *Culex declarator* Dyar & Knab, 1906 | ST11387, ST11395, ST11396 | M/L |
| *Culex mollis* Dyar & Knab, 1906 | ST11385, ST11386 | M |
| *Culex nigripalpus* Theobald, 1901 | MB10225, MB10226, MB10227, MB20197, MB20198 | L |
| *Culex quinquefasciatus* Say, 1823 | MB10496, MB10499, ST11390, ST11391 | M/F/L |
| *Culex surinamensis* Dyar, 1918 | ST11397, ST11398 | L |
| *Culex usquatus* Dyar, 1918 | MB10046, MB10049, ST10322, ST10323, ST10326, ST11399, ST11400 | M/F/L |
| **Subgenus *Melanoconion*** | | |
| *Culex abonnenci* Clastrier, 1970 | ST11437, ST11438, ST11439 | M |
| *Culex adamesi* Sirivanakarn & Galindo, 1980 | ST11326, ST11335 | M |
| *Culex alinkios* Sallum & Hutchings, 2003 | ST11309, ST11753, ST11755 | M/F |
| *Culex aphyllus* Talaga, 2021 | ST11551, ST11574 | M |
| *Culex bastagarius* Dyar & Knab, 1906 | ST11294, ST11296, ST12114 | M |
| *Culex batesi* Rozeboom & Komp, 1948 | ST11542 | M |
| *Culex bibulus* Dyar, 1920 | ST11297, ST11942, ST12110 | M |
| *Culex brachiatus* Hutchings & Sallum, 2008 | ST11331 | M |
| *Culex carincii* Talaga & Duchemin, sp. nov. | MB20290, MB20296 | M |
| *Culex caudatus* Clastrier, 1970 | ST12102 | M |
| *Culex caudelli* (Dyar & Knab, 1906) | ST11751, ST11752, ST11756 | M |
| *Culex clarki* Evans, 1924 | ST11922, ST11925 | M |
| *Culex comatus* Senevet & Abonnenc, 1939 | ST11295, ST11301, ST11313 | M |
| *Culex commevynensis* Bonne-Wepster & Bonne, 1920 | ST11343, ST12097, ST12103 | M |
| *Culex comminutor* Dyar, 1920 | MB10030, MB10806, MB10807, ST10669 | M/F/L |
| *Culex contei* Duret, 1968 | MB20273, MB20298, ST11918 | M |
| *Culex corentynensis* Dyar, 1920 | ST11701, ST11745 | M |
| *Culex creole* Anduze, 1949 | ST11307, ST11341, ST11712, ST11714 | M |
| *Culex cristovaoi* Duret, 1968 | ST11543 | M |
| *Culex dunni* Dyar, 1918 | MB20279, MB20281, MB20282 | M |
| *Culex eastor* Dyar, 1920 | MB20226, MB20242, MB20274, MB20283 | M |
| *Culex eknomios* Forattini & Sallum, 1992 | ST11544, ST11545, ST11555 | M |
| *Culex ensiformis* Bonne-Wepster & Bonne, 1920 | ST10672 | M |
| *Culex epanastasis* Dyar 1922 | ST11573, ST11703 | M |
| *Culex equinoxialis* Floch & Abonnenc, 1945 | ST11932, ST12003 | M |
| *Culex ernanii* Duret, 1968 | ST11852, ST11855, ST11945 | M |
| *Culex ernsti* Anduze, 1949 | ST11917, ST12190, ST12197, ST12198 | M |
| *Culex erraticus* (Dyar & Knab, 1906) | ST11923, ST11924, ST11926 | M |
| *Culex evansae* Root, 1927 | ST11984, ST12157 | M |
| *Culex extenuatus* Talaga & Duchemin, sp. nov. | ST11302, ST11308 | M |
| *Culex flabellifer* Komp, 1936 | ST11339, ST11340, ST11989 | M |
| *Culex foliafer* Komp & Rozeboom, 1951 | ST11315, ST11316 | M |
| *Culex galindoi* Komp & Rozeboom, 1951 | ST11708 | M |
| *Culex hutchingsae* Talaga, 2022 | ST11727, ST11729, ST11734 | M |
| *Culex idottus* Dyar, 1920 | ST11321, ST11322, ST11913 | M |
| *Culex inadmirabilis* Dyar, 1928 | ST11849, ST11850, ST11851 | M |
| *Culex innovator* Evans, 1924 | ST11317, ST11319, ST11336 | M |
| *Culex johnnyi* Duret, 1968 | ST11881, ST11882, ST11883 | M/F |
| *Culex johnsoni* Galindo & Mendez, 1961 | ST11577, ST11578, ST11579 | M |
| *Culex longistriatus* Sá & Hutchings, 2022 | ST12191, ST12192, ST12193, ST12195, ST12199 | M |
| *Culex lucackermanni* Talaga, 2022 | ST11742, ST11743, ST11744 | M |
| *Culex lucifugus* Komp, 1936 | ST10511, ST11116, ST11128 | M |
| *Culex ocossa* Dyar & Knab, 1919 | ST11914, ST11916, ST11919 | M |
| *Culex organaboensis* Talaga & Duchemin, 2023 | ST11977, ST11982 | M |
| *Culex pedroi* Sirivanakarn & Belkin, 1980 | MB20231, MB20289 | M |
| *Culex phlogistus* Dyar, 1920 | ST10656, ST10657, ST11990 | M |
| *Culex pilosus* (Dyar & Knab, 1906) | ST11211, ST11214, ST11324 | M |
| *Culex portesi* Senevet & Abonnenc, 1941 | MB20221, ST11354, ST11531 | M |
| *Culex productus* Senevet & Abonnenc, 1939 | ST11033, ST11035, ST11073 | M |
| *Culex putumayensis* Matheson, 1934 | MB20234, ST10180, ST11300, ST11312 | M/F |
| *Culex rabanicolus* Floch & Abonnenc, 1946 | MB20275, MB20299, ST11349 | M |
| *Culex rabelloi* Forattini & Sallum, 1987 | ST10832, ST11345, ST11696 | M |
| *Culex rorotaensis* Floch & Abonnenc, 1946 | ST10704, ST10705, ST11293 | M |
| *Culex sallumae* Talaga, 2022 | ST11700, ST11702, ST11704 | M |
| *Culex saramaccensis* Bonne-Wepster & Bonne, 1920 | ST11332 | M |
| *Culex serratimarge* Root, 1927 | ST11692, ST11693, ST11694 | M |
| *Culex simulator* Dyar & Knab, 1906 | ST11123, ST11124 | F |
| *Culex spinifer* Sá & Sallum, 2020 | ST11344 | M |
| *Culex spissipes* (Theobald, 1903) | MB20232, ST10637, ST12194 | M |
| *Culex taeniopus* Dyar & Knab, 1907 | ST12168 | F |
| *Culex theobaldi* (Lutz, 1904) | MB20055, ST10711, ST11275, ST11338 | M |
| *Culex tournieri* Senevet & Abonnenc, 1939 | ST11175, ST11342, ST11347 | M |
| *Culex unicornis* Root, 1928 | ST10714, ST10717, ST10727 | M |
| *Culex vaxus* Dyar, 1920 | ST10828, ST11272 | M |
| *Culex vomerifer* Komp, 1932 | ST11306, ST12080, ST12081 | M/F |
| *Culex ybarmis* Dyar, 1920 | ST11159, ST11711, ST11713 | M |
| *Culex zabanicus* Talaga & Duchemin, 2023 | ST11993, ST11997 | M |
| *Culex zeteki* Dyar, 1918 | ST11271 | M |
| **Subgenus *Microculex*** | | |
| *Culex pleuristriatus* Theobald, 1903 | MB10159, MB10166 | M/F |
| *Culex stonei* Lane & Whitman, 1943 | MB10154, MB10156, MB10240 | M/F/L |
| **Subgenus *Phenacomyia*** |  |  |
| *Culex corniger* Theobald, 1903 | ST11381, ST11382 | M |
| **Subgenus *Tinolestes*** | | |
| *Culex breviculus* Senevet & Abonnenc, 1939 | ST10946 | M |
| *Culex cauchensis* Floch & Abonnenc, 1945 | ST10668, ST11152 | M |
| **Without subgenus placement** | | |
| *Culex nigrimacula* Lane & Whitman, 1943 | MB10236, MB10237, MB10238 | L |
| *Culex ocellatus* Theobald, 1903 | MB10246, MB10247, MB10248, ST10187, ST10201 | M/F/L |
