# Supplementary material for: A DNA barcode library for Culex mosquitoes (Diptera: Culicidae) of South America with the description of two cryptic species of subgenus Melanoconion
Source: PLoS One. 2025 Feb 21;20(2):e0310571. doi: 10.1371/journal.pone.0310571 (PMC11845035; doi:10.1371/journal.pone.0310571)
Supplement: S3 Table — Species are listed alphabetically by subgenus except when more than one morphological species are included in a BIN. Distances (p-distance) correspond to the percentage of dissimilar pairwise nucleotides and counts correspond to the number of voucher specimens included in this study followed, between brackets, by the total number of specimens (including ours) present in the BOLD database. (DOCX) [file pone.0310571.s003.docx]

**S2 Table List of Barcode Index Numbers (BINs) with their associated *Culex* species obtained from BOLD (last visited august 2024).** Species are listed alphabetically by subgenus except when more than one morphological species are included in a BIN. Distances (p-distance) correspond to the percentage of dissimilar pairwise nucleotides and counts correspond to the number of voucher specimens included in this study followed, between brackets, by the total number of specimens (including ours) present in the BOLD database.

| **BIN** | **Species** | **Average distance** | **Maximum distance** | **Nearest-neighbor distance** | **Count** |
| --- | --- | --- | --- | --- | --- |
| **Subgenus *Aedinus*** | | | | | |
| ABY1528 | *Culex accelerans* | 1.28 | 1.93 | 8.35 | 2(3) |
| AAU2664 | *Culex amazonensis* | 1.19 | 2.89 | 8.35 | 2(21) |
| **Subgenus *Anoedioporpa*** | | | | | |
| ACZ4266 | *Culex originator* | 0.77 | 1.28 | 2.07 | 5 |
| **Subgenus *Carrollia*** | | | | | |
| AEE3991 | *Culex antunesi* | 0.21 | 0.32 | 8.67 | 3 |
| AAW1433 | *Culex bonnei* | 0.29 | 0.48 | 4.33 | 2(6) |
| ACZ3921 | *Culex secundus* | 0.35 | 0.64 | 4.33 | 3(4) |
| AAG3837 | *Culex urichii* | 0.4 | 0.76 | 7.87 | 5(14) |
| **Subgenus *Culex*** | | | | | |
| AAW1267 | *Culex bonneae* | 0.39 | 0.96 | 6.04 | 4(5) |
| AAN3636 | *Culex brevispinosus* | 1.4 | 3.68 | 2.75 | 2(464) |
|  | *Culex surinamensis* |  |  |  | 2(464) |
|  | *Culex usquatus* |  |  |  | 7(464) |
| AAF1735 | *Culex declarator* | 1.45 | 5.07 | 1.59 | 3(926) |
|  | *Culex mollis* |  |  |  | 2(926) |
|  | *Culex nigripalpus* |  |  |  | 5(926) |
| AAA4751 | *Culex quinquefasciatus* |  |  |  | 4(6310) |
| **Subgenus *Melanoconion*** | | | | | |
| AEE5994 | *Culex abonnenci* | 0 | 0 | 1.44 | 3 |
| AEE6675 | *Culex adamesi* | 0.16 | 0.16 | 2.41 | 2 |
| ADJ7929 | *Culex alinkios* | 0.41 | 1.12 | 1.96 | 3(7) |
| AFI9537 | *Culex aphyllus* | 0.48 | 0.48 | 4.17 | 2 |
| AFI9514 | *Culex bastagarius* | NA | NA | 4.98 | 1 |
| AEE3102 | *Culex bastagarius* | 0 | 0 | 5.14 | 2 |
| ADK0770 | *Culex batesi* | 1.25 | 1.77 | 6.58 | 1(5) |
|  | *Culex evansae* |  |  |  | 2(5) |
| AEE1543 | *Culex bibulus* | 1.12 | 1.12 | 2.41 | 2 |
| AFI9809 | *Culex bibulus* | NA | NA | 2.41 | 1 |
| AEE7320 | *Culex brachiatus* | 0.11 | 0.16 | 2.09 | 1(4) |
| AEE7955 | *Culex carincii* | 1.34 | 1.44 | 2.25 | 2(3) |
| AFI9952 | *Culex caudatus* | NA | NA | 4.33 | 1 |
| AFI9953 | *Culex caudelli* | 1.61 | 1.93 | 5.94 | 3 |
| ADK1664 | *Culex clarki* | 1.77 | 1.93 | 7.87 | 2(4) |
| AEE7402 | *Culex comatus* | 0.21 | 0.32 | 3.21 | 3 |
| ADK0771 | *Culex commevynensis* | 1.06 | 1.77 | 4.82 | 3(5) |
| ACZ4398 | *Culex comminutor* | 0.43 | 0.64 | 6.1 | 4 |
| AEE2103 | *Culex contei* | 1.46 | 2.09 | 3.85 | 3(10) |
|  | *Culex phlogistus* |  |  |  | 3(10) |
|  | *Culex serratimarge* |  |  |  | 3(10) |
| AFI9596 | *Culex corentynensis* | 0.96 | 0.96 | 5.94 | 2 |
| AEE6759 | *Culex creole* | 0.66 | 1.77 | 4.26 | 4(20) |
|  | *Culex eastor* |  |  |  | 4(20) |
|  | *Culex hutchingsae* |  |  |  | 3(20) |
|  | *Culex idottus* |  |  |  | 3(20) |
| AFI9595 | *Culex cristovaoi* | NA | NA | 8.67 | 1 |
| AEE2793 | *Culex dunni* | 0.96 | 1.12 | 2.09 | 3 |
| AFI9981 | *Culex eknomios* | 0 | 0 | 1.12 | 3 |
| ADJ7931 | *Culex ensiformis* | 2.09 | 2.09 | 2.73 | 1(2) |
| AFI9300 | *Culex epanastasis* | 0.32 | 0.32 | 6.42 | 2 |
| ADK1666 | *Culex equinoxialis* | 1.19 | 1.77 | 8.35 | 2(5) |
| AFI9301 | *Culex ernanii* | 0.21 | 0.32 | 7.17 | 3 |
| AFB1366 | *Culex ernsti* | 0.99 | 1.61 | 3.69 | 4 |
| AAG3848 | *Culex erraticus* | 2.06 | 3.69 | 1.93 | 3(86) |
| ADK4497 | *Culex extenuatus* | 1.09 | 2.57 | 4.01 | 2(14) |
| AEE1183 | *Culex flabellifer* | 0.32 | 0.48 | 2.73 | 3 |
| AEE1181 | *Culex foliafer* | NA | NA | 4.33 | 1 |
| AEE1182 | *Culex foliafer* | NA | NA | 5.14 | 1 |
| AFI9826 | *Culex galindoi* | NA | NA | 8.51 | 1 |
| AFJ0561 | *Culex inadmirabilis* | NA | NA | 2.41 | 1 |
| AFJ0562 | *Culex inadmirabilis* | 1.93 | 1.93 | 2.41 | 2 |
| ABZ4907 | *Culex innovator* | 1.32 | 3.05 | 1.99 | 3(20) |
| AFI9771 | *Culex johnnyi* | 0.75 | 0.96 | 4.65 | 3 |
| AFI9770 | *Culex johnsoni* | 0.75 | 0.8 | 7.21 | 3 |
| AFI9808 | *Culex longistriatus* | 0 | 0 | 6.42 | 5 |
| AET1273 | *Culex lucackermanni* | 0 | 0 | 3.21 | 3 |
| ACU4075 | *Culex lucifugus* | 0.92 | 1.62 | 2.41 | 3(20) |
| AAG3846 | *Culex ocossa* | 0.81 | 4.56 | 2.61 | 3(24) |
| AFJ0416 | *Culex organaboensis* | NA | NA | 2.09 | 1(2) |
| AFT5569 | *Culex organaboensis* | NA | NA | 2.09 | 1(2) |
| AAI9888 | *Culex pedroi* | 1.17 | 2.25 | 6.58 | 2(10) |
| AAG3858 | *Culex pilosus* | 1.29 | 1.61 | 2.61 | 3(4) |
| ACS6189 | *Culex portesi* | 0.09 | 0.32 | 3.7 | 3(7) |
| AEE7867 | *Culex productus* | 1.07 | 1.61 | 6.1 | 3 |
| ACZ3899 | *Culex putumayensis* | 0.32 | 0.64 | 3.37 | 4 |
| AEE2997 | *Culex rabanicolus* | 1.07 | 1.28 | 2.73 | 3 |
| ADE6009 | *Culex rabelloi* | 1.12 | 1.28 | 1.25 | 2(3) |
| AEW5154 | *Culex rabelloi* | 0.24 | 0.36 | 1.25 | 1(3) |
| AEE5422 | *Culex rorotaensis* | 0.21 | 0.32 | 6.61 | 3 |
| AER7739 | *Culex sallumae* | 0 | 0 | 8.99 | 3 |
| AEE1449 | *Culex saramaccensis* | NA | NA | 7.22 | 1 |
| AEE8544 | *Culex simulator* | 1.12 | 1.12 | 8.83 | 2 |
| AEE6947 | *Culex spinifer* | NA | NA | 8.51 | 1 |
| ABY1758 | *Culex spissipes* | 1.28 | 2.57 | 2.69 | 3(13) |
| AFI9705 | *Culex taeniopus* | NA | NA | 9.31 | 1 |
| ADK5539 | *Culex theobaldi* | 1.17 | 1.77 | 2.25 | 3(6) |
| AEE9553 | *Culex theobaldi* | NA | NA | 2.25 | 1 |
| AEE9698 | *Culex tournieri* | 0.62 | 0.8 | 1.77 | 3 |
| AEE9682 | *Culex unicornis* | 1.18 | 1.28 | 4 | 3 |
| ADJ7555 | *Culex vaxus* | 0.75 | 1.12 | 2.89 | 2(3) |
| ADT9229 | *Culex vomerifer* | 1.5 | 2.89 | 6.9 | 3(13) |
| AEE1897 | *Culex ybarmis* | 0.11 | 0.16 | 2.89 | 3 |
| AFI9750 | *Culex zabanicus* | 0 | 0 | 7.7 | 2(4) |
| AGA9331 | *Culex zeteki* | NA | NA | 2.09 | 1(1) |
| **Subgenus *Microculex*** | | | | | |
| ACZ4187 | *Culex pleuristriatus* | 0.96 | 1.44 | 7.5 | 2(4) |
| ACZ4071 | *Culex stonei* | 0.2 | 0.31 | 4.65 | 3 |
| **Subgenus *Phenacomyia*** | | | | | |
| ADV2314 | *Culex corniger* | 0.12 | 0.17 | 1.4 | 2(3) |
| **Subgenus *Tinolestes*** | | | | | |
| AEE2377 | *Culex breviculus* | NA | NA | 7.06 | 1 |
| AEE4112 | *Culex cauchensis* | 0.16 | 0.16 | 7.7 | 2 |
| **Without subgenus placement** | | | | | |
| ACZ4194 | *Culex nigrimacula* | 0 | 0 | 9.15 | 3 |
| ACZ4158 | *Culex ocellatus* | 0.43 | 0.92 | 8.82 | 5 |
