# Supplementary figures and images for: A DNA barcode library for Culex mosquitoes (Diptera: Culicidae) of South America with the description of two cryptic species of subgenus Melanoconion
Source: PLoS One. 2025 Feb 21;20(2):e0310571. doi: 10.1371/journal.pone.0310571 (PMC11845035; doi:10.1371/journal.pone.0310571)

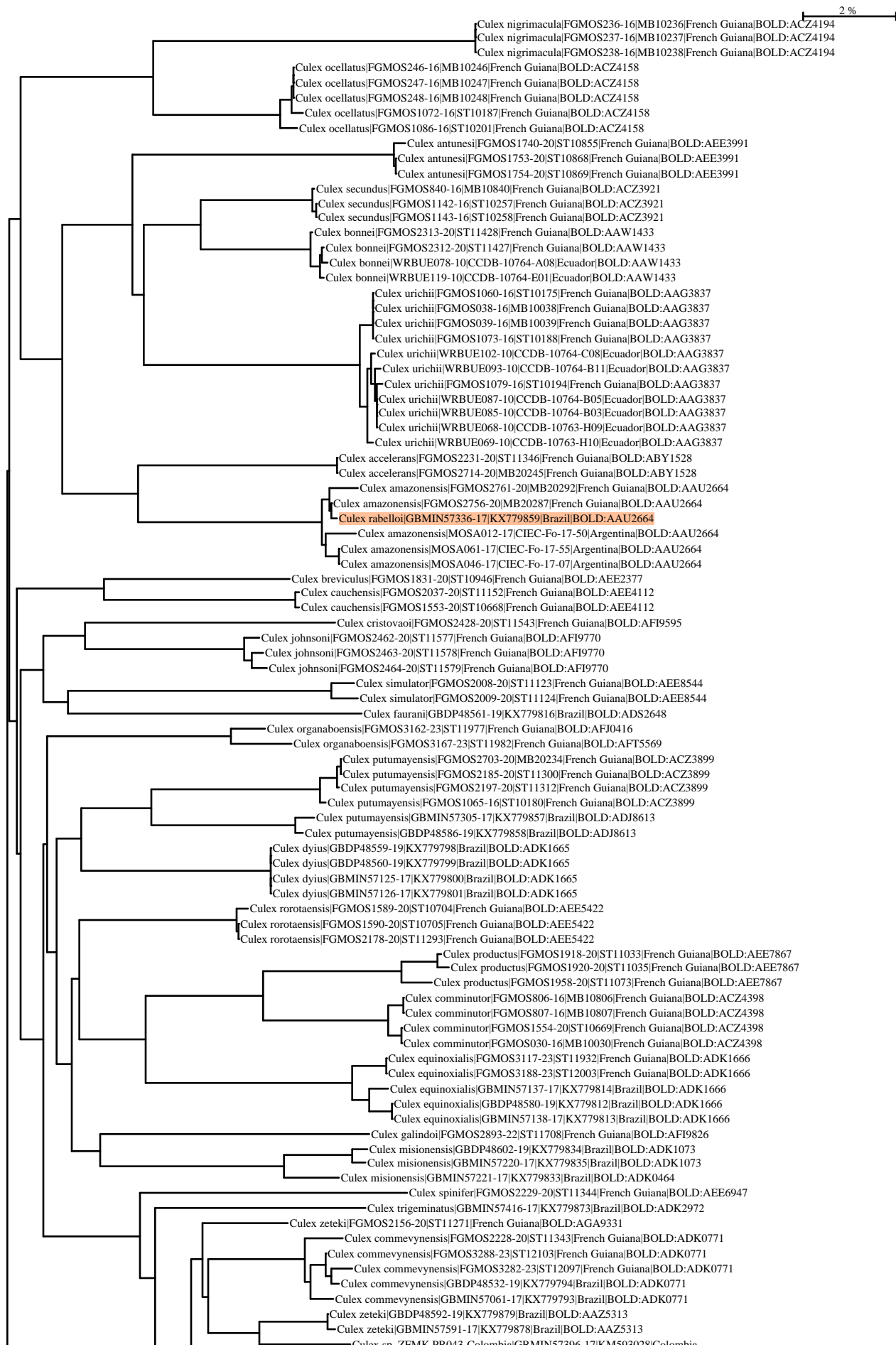

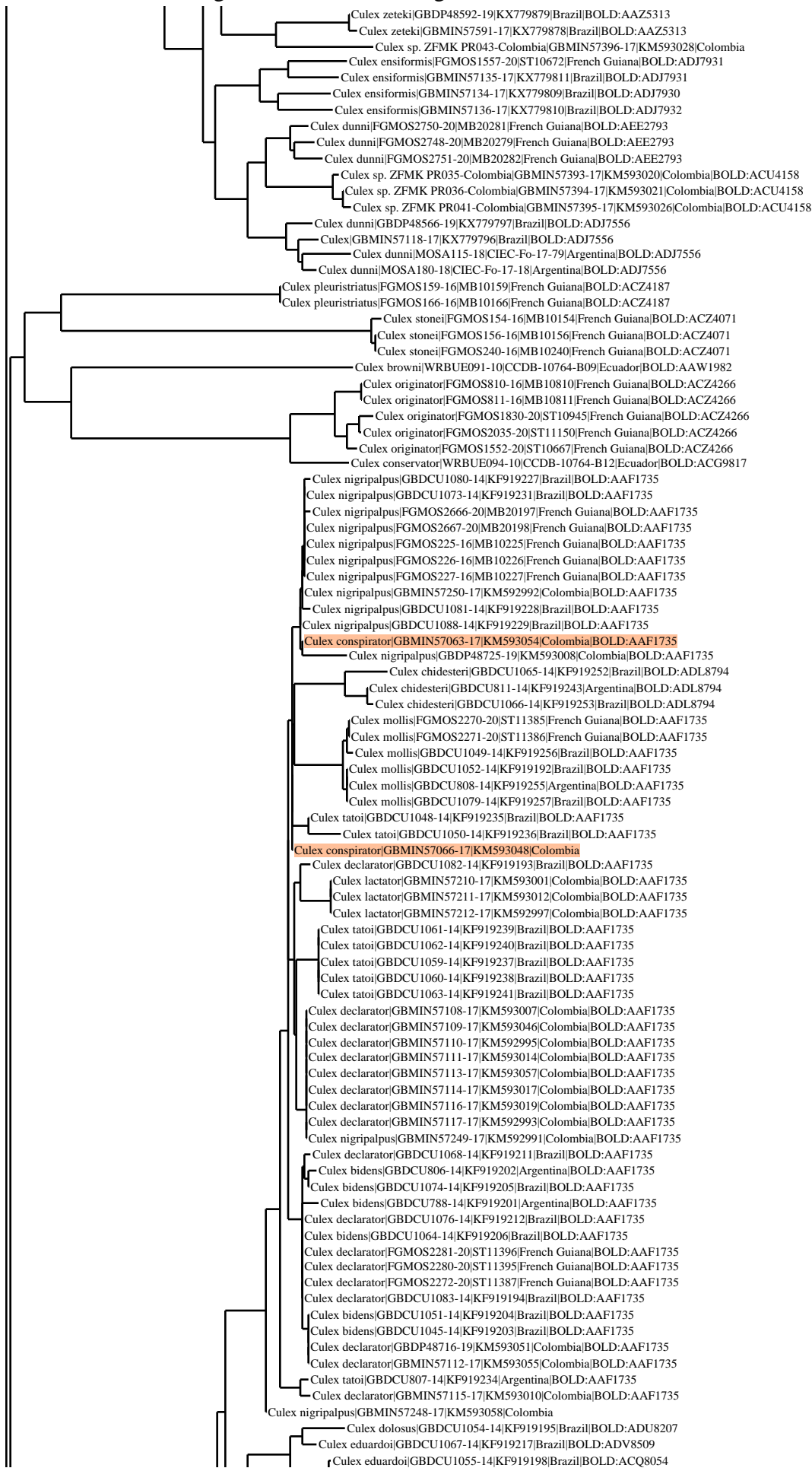

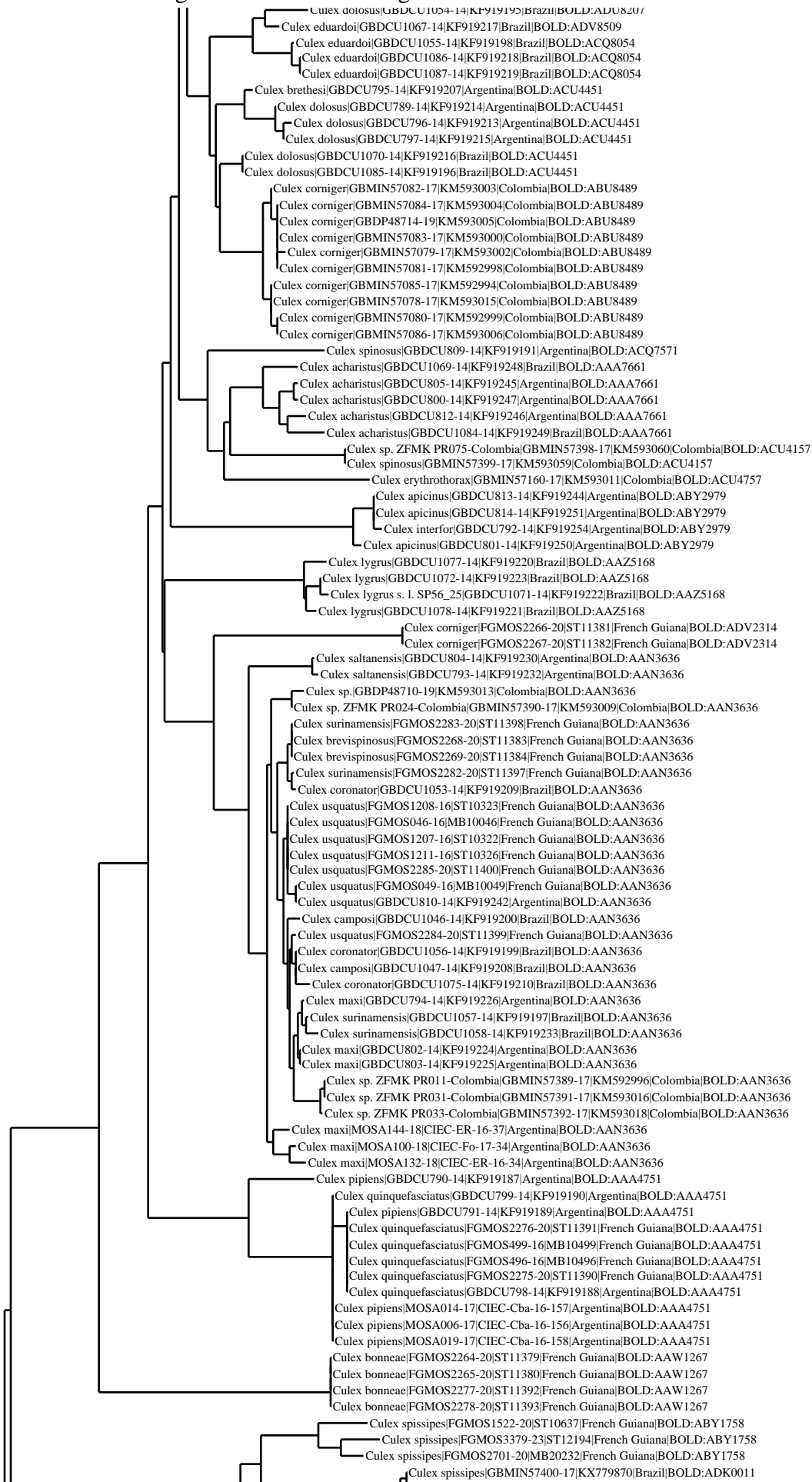

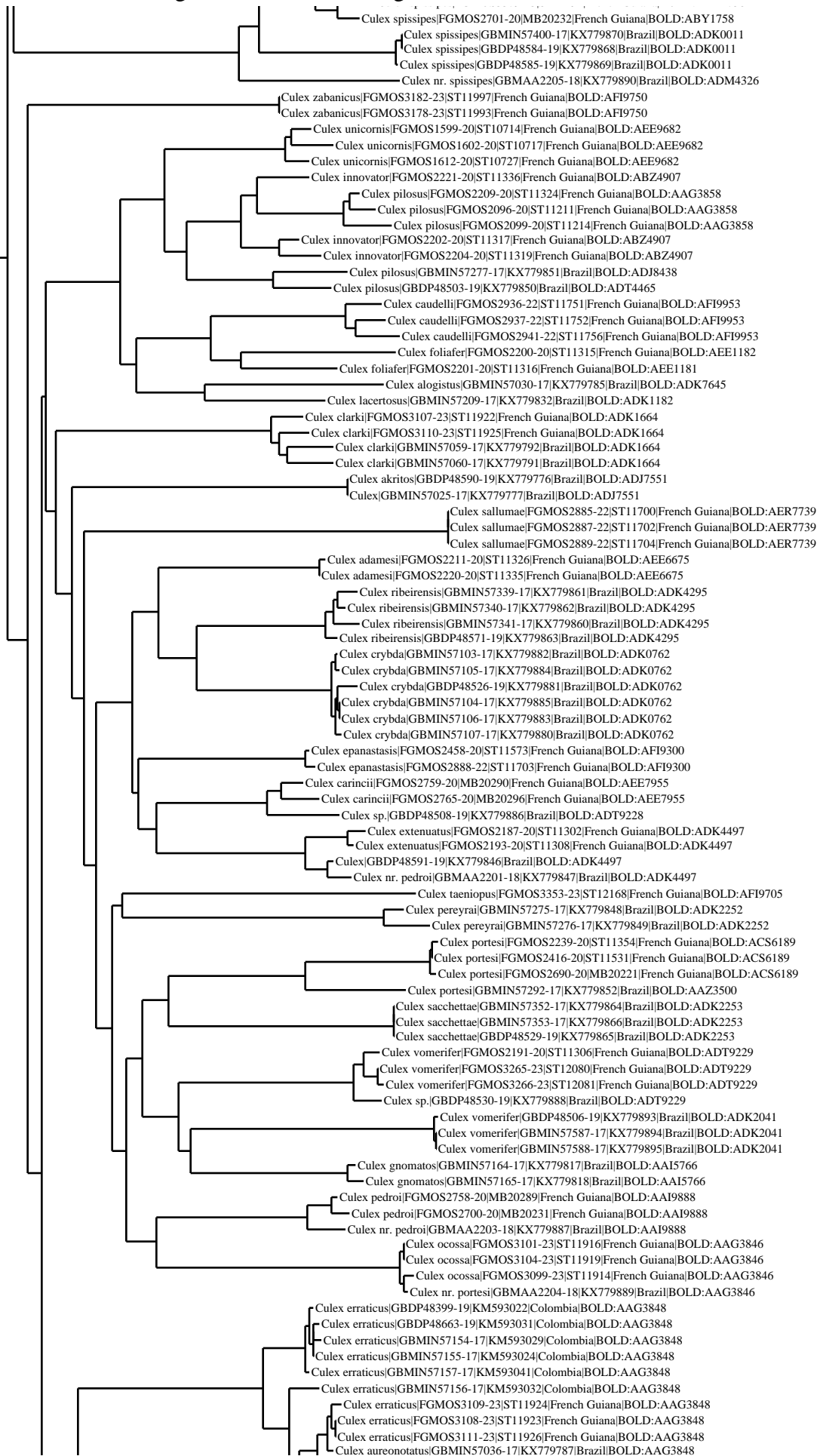

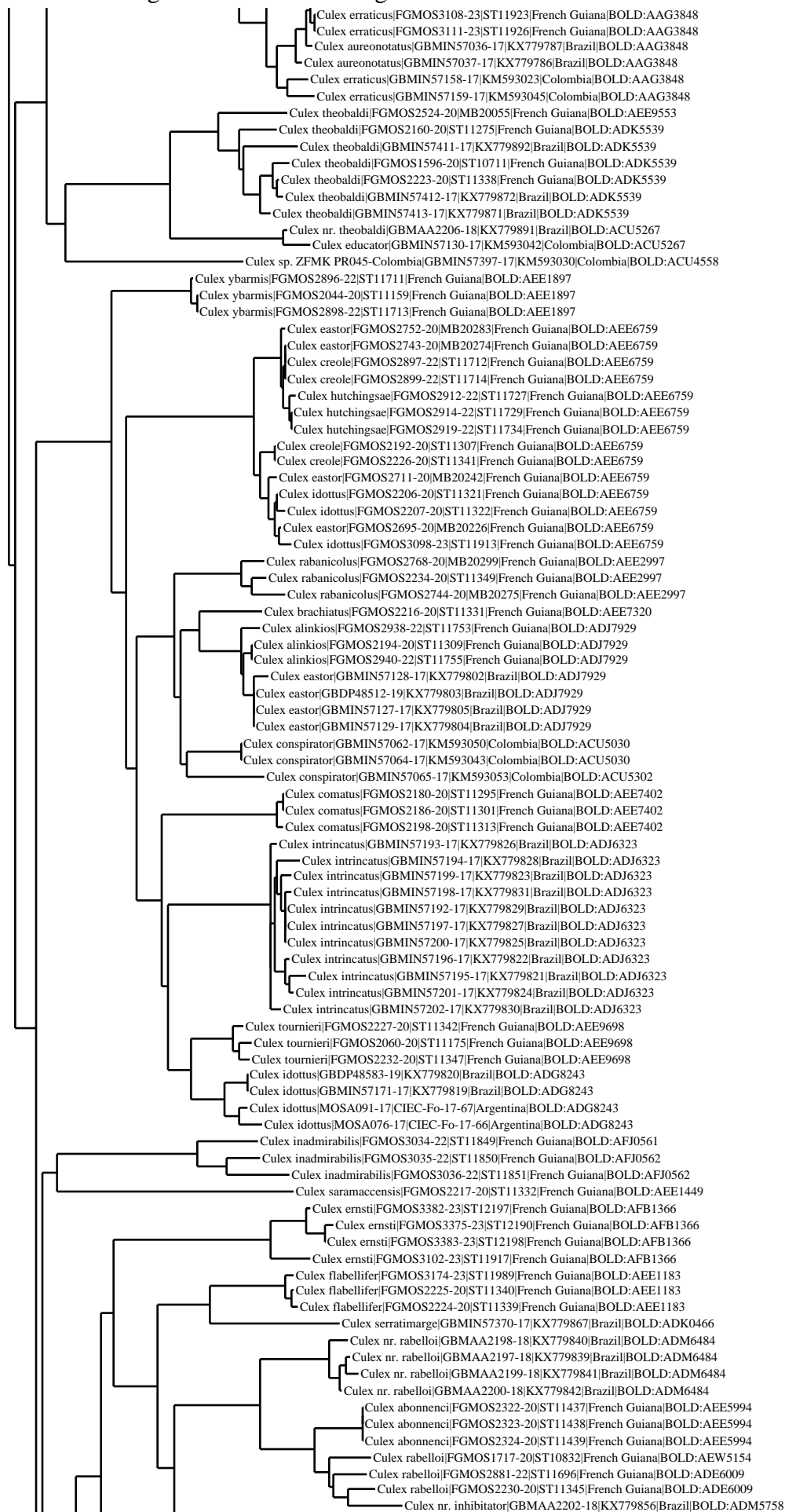

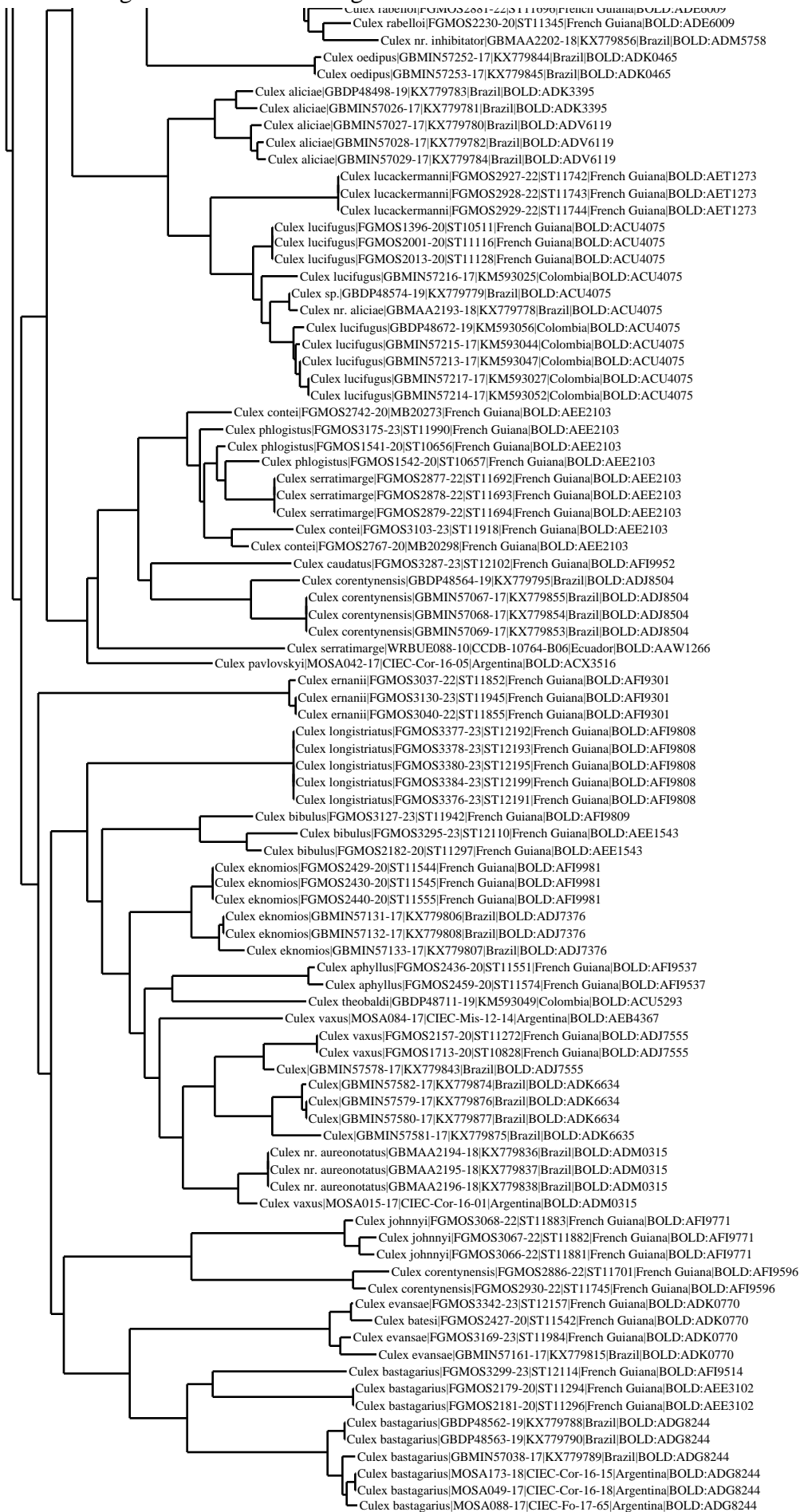

Supplement: S1 Fig — This dataset is composed of 246 sequences from French Guiana, 164 sequences from Brazil [17; 21], 62 sequences from Colombia [20], 46 sequences from Argentina [17; 22], and 11 sequences from Ecuador [16]. For each specimen, we indicated the morphological identification, the BOLD specimen code, the original specimen code, the sampling country and the BOLD Barcode Index Number (BIN). Identification of eight specimens from Brazil were missing in BOLD. Identifications of these specimens in the original publication [21] were as follows: KX779777: Culex akritos, KX779846: Culex nr. pedroi, KX779796: Culex dunni, and KX779874–KX779877 and KX779843: Culex vaxus. (PDF) [file pone.0310571.s004.pdf]
